# Perspectives in the Computational Modeling of New Generation, Biocompatible Ionic Liquids

**DOI:** 10.1021/acs.jpcb.1c09476

**Published:** 2022-01-03

**Authors:** Enrico Bodo

**Affiliations:** Chemistry Department, University of Rome “La Sapienza”, P. A. Moro 5, 00185 Rome, Italy

## Abstract

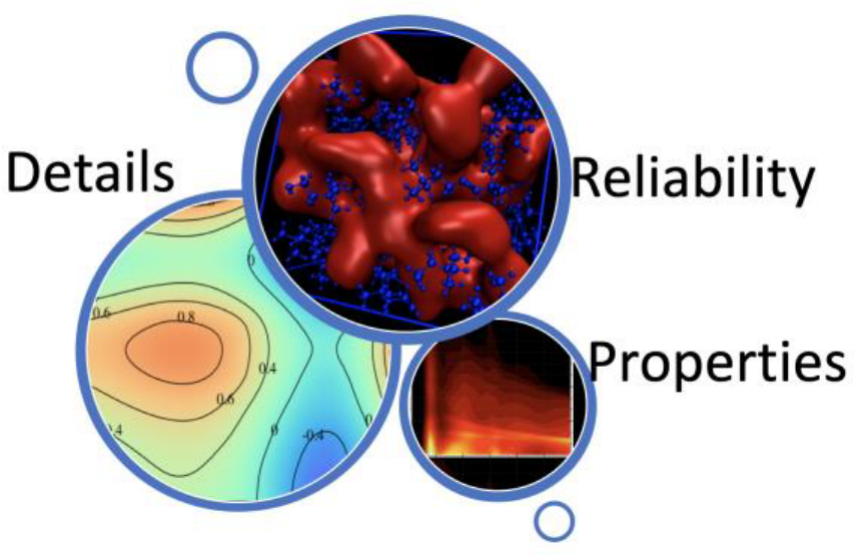

In this Perspective, I review the current state of computational simulations on ionic liquids with an emphasis on the recent biocompatible variants. These materials are used here as an example of relatively complex systems that highlights the limits of some of the approaches commonly used to study their structure and dynamics. The source of these limits consists of the coexistence of nontrivial electrostatic, many-body quantum effects, strong hydrogen bonds, and chemical processes affecting the mutual protonation state of the constituent molecular ions. I also provide examples on how it is possible to overcome these problems using suitable simulation paradigms and recently improved techniques that, I expect, will be gradually introduced in the state-of-the-art of computational simulations of ionic liquids.

## 1 Introduction

Few inventions have impacted society and especially societal relations as the computer has done. The recent diffusion of easily portable computers in the form of smartphones and other devices has altered the way in which people communicate and has made possible the emergence of a new form of society (unthinkable just few decades ago) where the dissemination of information is immediate and global. The same revolution has impacted science and the way in which it is communicated or divulged. A less evident (at least outside the scientific community), but likewise innovative revolution has impacted the way in which science is done: a revolution boosted by the impressive surge in computer power and by the ever-increasing availability of computers.

The importance of computational approaches in science can be traced back few decades by just highlighting some of the past Nobel prizes. It starts with Fukui and Mulliken and the work of Hauptman and Karle and continues in 1998 with the prize to Kohn and Pople and, even more recently, with the recognition of the pioneering work of Karplus, Levitt, and Warshel in 2013. The 2021 Nobel prize has been awarded to Manabe and Hasselmann for “for the physical modelling of Earth’s climate” and to Parisi, who was involved in the birth of APE supercomputers in early nineties, for his work on complex systems. Other important achievements in human knowledge have been reached through the intensive use of computer and computations, just to name two: the measurement of the rate of the expansion of the Universe and the mapping of the human genome.

As implicitly recognized by the short and incomplete list above, chemistry is one of the fields that has benefitted most from the introduction of computers and their use. The application of computations to chemistry has spawned an entire new discipline aptly named “computational chemistry”. Under this umbrella we find the widest field of activities ranging from biology to molecular physics. This means that computational chemistry concerns a huge range of different systems, from the micro to the nano scale, from millions of atoms (e.g., proteins) to few atom systems (isolated molecules). The study of molecular systems from a fundamental point of view through computer models, has consequences in high societal impact areas such as climate changes, green economy, medicine, etc.

Despite the huge improvements in computer performances and in methodologies, the modeling and simulation of complex and heterogeneous molecular systems is still a challenging, often frustrating, task in chemistry. Notwithstanding, the opportunity to provide reliable data using a computer model has been and will be a great advantage for an efficient design of laboratory activities. It is sufficient to think that a well-designed set of computational simulations can avoid tedious and repetitive experimental procedures, thereby allowing the research to focus on the most promising materials or processes for a given aim. The realization of this scenario obviously depends on the reliability of the computer models, a question that is now central to the computational chemistry community and that is the subject of this article.

Computational chemistry consists of a wide array of techniques and methods which have their roots and foundation in physics, in the molecular Schrodinger equation (SE, mainly in its nonrelativistic form). In principle, the SE tells us how a given physical quantum system evolves in time. As is well-known, solving the SE for the quantum evolution of a molecular system of a decent complexity (let us say more than five atoms) is impractical or simply impossible. Computational chemistry, using Dirac’s words, consists of the development and use of “*approximate practical methods of applying quantum mechanics [···] which can lead to an explanation of the main features of complex atomic systems without too much computation*”.

Two of these approximate methods are the cornerstone of modern computational chemistry: the Born–Oppenheimer (BO) approximation and the classical motion of the nuclei. The former allows the calculation of electronic energies and the use of these as the source of the forces that act on the nuclei, the latter permits to blend nuclei into “classical” atoms for the implementation of simple and inexpensive methods to compute the evolution of complex chemical systems.

The BO approximation can be formulated for all molecular systems and states that the nuclei move under the effect of forces that are the gradient of the electronic energies computed at fixed nuclear geometries. This, effectively, decouples the solution of the nuclear and electronic SE in a two-step procedure: (i) first, one computes the electronic energy as a function of the nuclear geometries, and (ii) then, one solves the nuclear motion using the electronic energy as the potential. The computational techniques for the first step are well-known and are based on either wave function-based methods (Hartree–Fock or post-Hartree–Fock calculations)^1,2^ or Density Functional Theory (DFT).^3^ Both approaches are often called ab initio methods because they do not employ empirical parameters.

Essentially, operating under the BO approximations consists of assuming that the electrons are instantaneously in a stationary state at a given nuclear geometry and that the couplings due to the simultaneous motion of nuclei and electrons are negligible. In practice, this is achieved by initially fixing the position of the nuclei (an approach known as *clamped* nuclei) and solving the resulting electronic SE where the nuclear kinetic energy has been neglected. Such SE is solved through ab initio approaches to find an approximation to the ground state wave function (or energy) that governs the electronic motion subjected to the specific, geometry-dependent nuclear electrostatic potential. The ground state electronic energy (i.e., the eigenvalue of the electronic SE) depends on the nuclear geometry and is a function of the nuclear coordinates. As such, in the BO approximation, the electronic energy is interpreted as the potential (i.e., the source of the force) that governs the nuclear motion.

Chemistry is essentially the study of transformations that take place over a given time. It therefore follows that dynamic, i.e. the nuclear (atomic) motion, is the crucial issue. Electronic structure calculations such as DFT are inherently static, that is they provide an instantaneous “snapshot” of a given chemical system but yield only limited information about its chemical evolution. But it is precisely the evolution of the molecular motion that gives rise to chemical processes. Very often, these processes, especially when complex materials are involved, stem from emerging properties which do not simply appear to arise from the combination of those of the molecular constituents.^4^ Emerging patterns and complex properties surface because the simpler constituents behave collectively over a given time span in certain ways and not in others. This means that statistics and probability (i.e., entropy) play a crucial role in determining the outcome of a given chemical phenomena.

The problem with dynamic is that the quantum evolution of given chemical system of certain complexity is impossible to calculate. The trick that computational chemists use consists of exploiting the BO approximation and blend the nuclei into ideal classical objects whose quantum features can be neglected. In most applications, these objects are meant to be “atoms”, but, in principle, they can also represent other entities.^5^ This approach is broadly known as Molecular Dynamics (MD).^6^ MD is indeed based on treating the evolution of the atoms using Newton’s laws of motion where the forces are determined by the electronic energies. In this context, disregarding the quantum nature of the heavy particles produces a set of systematic errors that lie in neglecting zero-point-energies and tunnelling, neglecting coherence, symmetry, and exchange effects, and ignoring the discrete nature of the energies of the molecular motions. Even though some of these issues can actually be accounted for in some way,^7^ their inclusion is still missing in most traditional MD techniques.

When it comes to MD, another crucial issue is the origin and the accuracy of the forces that acts on the atoms. In principle one should use the gradient of the electronic energies obtained by solving the electronic SE, but often, in practice, one is limited by size and must resort to a rough parametrization known as the “force field”. The former approach is known as Born–Oppenheimer MD (BOMD) also called ab initio MD (AIMD).^8^ The latter consists of the so-called “classical” MD.^9^ The forces fields are built using simple analytical expressions that mimic the true quantum electronic potential. Generally, in these force fields, the intramolecular interactions are treated separately from the intermolecular ones. For example, a typical intermolecular atom–atom interaction is modeled using an expression that is the sum of a repulsive term (thus reproducing the exchange interaction at short nuclear distance) and an attractive one (that accounts for the long-range dispersive forces between atoms). The intramolecular forces are treated using elaborate analytical expressions that describe the covalently bound groups of atoms and give shape to the molecular structure.

The choice between BO and classical (force field) MD is dictated by the size of the systems and by the time scale along which the chemical events take place. For systems comprising thousands or more atoms and for time scales larger than a few hundreds of picoseconds, classical MD is the only option. For smaller systems and shorter time scales AIMD becomes feasible. Methods that require a less expensive way to solve the electronic Schrodinger equation, such as semiempirical approaches,^10,11^ can also be used to alleviate the computational costs associated with AIMD.

## 2 The Materials

As the title says, we focus the following discussion on biocompatible ionic liquids. These liquids are built using relatively simple molecular ions, but due to a surprisingly cooperative dynamics, show a range of complex phenomena that represent the optimal playground to illustrate some of the difficulties and challenges that theoretical and computational simulations will have to confront in the immediate future; especially if we desire to apply them to the increasingly complex problems that emerge from new technologies of societal relevance (new batteries, sustainable materials, new biomaterials, nanomedicine, etc.).

Ionic liquids (ILs) represent a significant opportunity for replacing, at least partly, the solvents currently used in various fields in industry, chemical synthesis, preservation, analysis, etc. To name a few, ILs have been proposed as solvents for electrochemical applications,^12,13^ corrosion inhibitors,^14^ catalysts,^15^ removal agents of polluting gases,^16−21^ and dissolving agents for biomasses.^22^

The impressive increase in recent years of the studies concerning ILs in the most varied field of research and technology, is also due to the fact that, for a long time, they have been considered inherently green replacements for traditional chemical solvents (often notoriously harmful to environment and living species). Their extremely low volatility and high chemical stability are indeed huge advantages in case of leaks or laboratory operations. However, recent studies have revisited the presupposed green nature of ILs and discovered that most of them are actually toxic toward organisms and less environmentally benign than previously assumed.^23−26^ Despite the large number of studies about the toxicity of traditional ILs, a molecular understanding of their action toward biomolecules is still largely missing.^23^

**Scheme 1:**
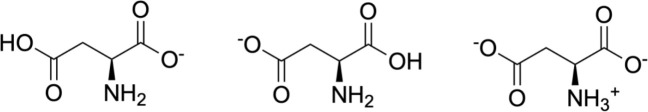
Isomers of the [Asp]^−^ Anion That May Be in Equilibrium inside the Bulk Phase of an AAILs

In the past few years, part of the research effort has steered toward the quest for truly biocompatible ILs. Typically, these liquids are obtained using the cholinium cation, a metabolic harmless substance, as a replacement of the imidazolium of traditional ILs.^27−29^ Deprotonated amino acids (see Scheme 1 and Figure 5) or simple organic acids (see Figure 6) are used as anions giving rise to the subgroups of ILs known respectively as amino acid based ILs (AAILs) and acid–based ILs (AILs). Formally, (A)AILs are obtained using a simple acid/base reaction, hence they pertain to the class of ILs called Protic ILs (PILs) where ionization occurs because of the following equilibrium
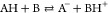
1where, to form a PIL, the reaction must be completely shifted toward the products, when all neutral components (the reagents) disappear.

ILs, in general, show a rather complicated pattern of structural features at the nanoscopic scale.^30^ This includes segregation phenomena,^31−33^ self-assembly,^34,35^ and unusual HB networks.^36−42^ PILs, in particular, are characterized by the existence of a pervasive network of hydrogen bonds (HBs) that makes them unique in the vast population of possible ILs. The presence of these HBs is the source of their peculiar solvation properties that has spawned a concrete interest from the biomedical community,^43,44^ for possible applications in circular economy,^45−47^ and in electrochemistry.^12,48,49^ This very same network of HB spans a range of nanoscopic phenomena that makes the study of these substances particularly intriguing from the computational point of view, but it also represents a challenge for current state-of-the-art techniques.

## 3 Methods: State-of-the-Art

**Figure 1:**
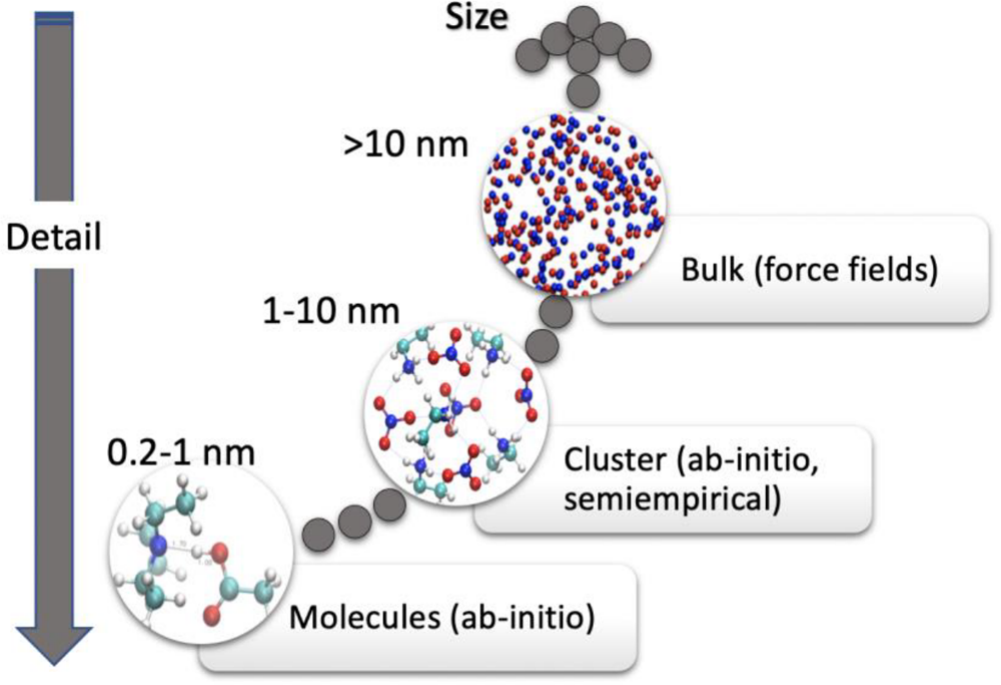
Sketch indicates the three system sizes typically explored by computational chemists in the simulations of ILs. A reasonable indication of the appropriate method is also reported.

The computer simulations of ILs are performed through a variety of techniques. These are generally chosen depending on the size of the simulated system. Roughly speaking one can divide the continuous size scale (from hundreds of picometers of isolate molecules well into the nanometer range of macromolecules) into three different realms (Figure 1): isolated molecules or dimers, clusters composed by a small number of molecules, and bulk. As shown in Figure 1, as the size of the system (i.e., the number of atoms or entities making up the simulation) increases, the level of detail which we obtain from the simulations tends to decrease. Although there might be significant exceptions, Figure 1 also illustrates the typical appropriate computational method for each size.

Typically, when moving from isolated molecules to clusters to bulk, one has to compromise (due to the computational cost) and loose a certain level of details. While *ab initio* electronic structure calculation such as those based on electronic wave functions or DFT are available at the molecular and cluster level, they rapidly become impractical when simulating the bulk phase that is the realm of methods based on the adoption of parametric forces (the *force fields*) where the details of the electronic structure and connected phenomena are lost. It is worth pointing out that the implicit hierarchy in Figure 1 is based exclusively on considerations of system size and details, but it does not necessarily entail any assumption about the accuracy of the methods involved. Often a force field-based MD can better match experiments than ab initio depending on how well the force field has been parametrized.

The advantage of an approach based on the study of isolated molecular entities or dimers is that all the details of the electronic and nuclear motions are accessible and that the accuracy of the calculation can be pushed to the boundary of what is possible today. The main drawbacks of such approach consist in the lack of many-body effects and in the impossibility of modeling all those patterns and phenomena that clearly emerge because of collective or cooperative molecular behaviors (segregation, assembly etc.). When instead one works at the bulk scale (disregarding the case of perfect crystals where periodicity translates into a simplified computational model), one loses the electronic details, but there is a chance of obtaining information on collective phenomena.

Between these two regimes, there is space for an approach based on the analysis of small aggregates of molecules or ions. Although this is a level only seldom explored, it represents the ideal approach for looking at how the molecular properties are gradually affected by an increased presence of a surrounding. The advantage of an approach based on a cluster is that highly detailed calculations are still doable together with the possibility of implementing nontrivial dynamic analysis as well. Its drawbacks substantially lie in the inevitable presence of border effects due to the finite size of the clusters.

It is rather evident from the above discussion that to sample the behavior of relatively complex materials such as ILs, considering the “dynamic” factor is essential to understand the origin of their properties and to provide predictive and reliable models on which the planning of new technologies could be implemented. For this reason, I will concentrate mainly on dynamics in the rest of the paper.

The aims of performing MD on ILs is manyfold: first and foremost, it is needed to accumulate a set of configurations that should span the dominant regions of the conformational (phase) space of the system; this, in turn, allows the calculation of thermodynamic functions (energy, enthalpy, free energy, molar volume, etc.) using time or ensemble averages using statistical mechanics. Second, MD can be used to study the structural features of the system, albeit in an averaged fashion (X-ray diffraction patterns, for example). Finally, it provides the frictional properties of the liquid (viscosities, conductivities, diffusion etc.) which are linked to the kinetic of the molecular motion inside fluid itself, with these typically obtained using Green–Kubo relations.^50−52^

**Figure 2:**
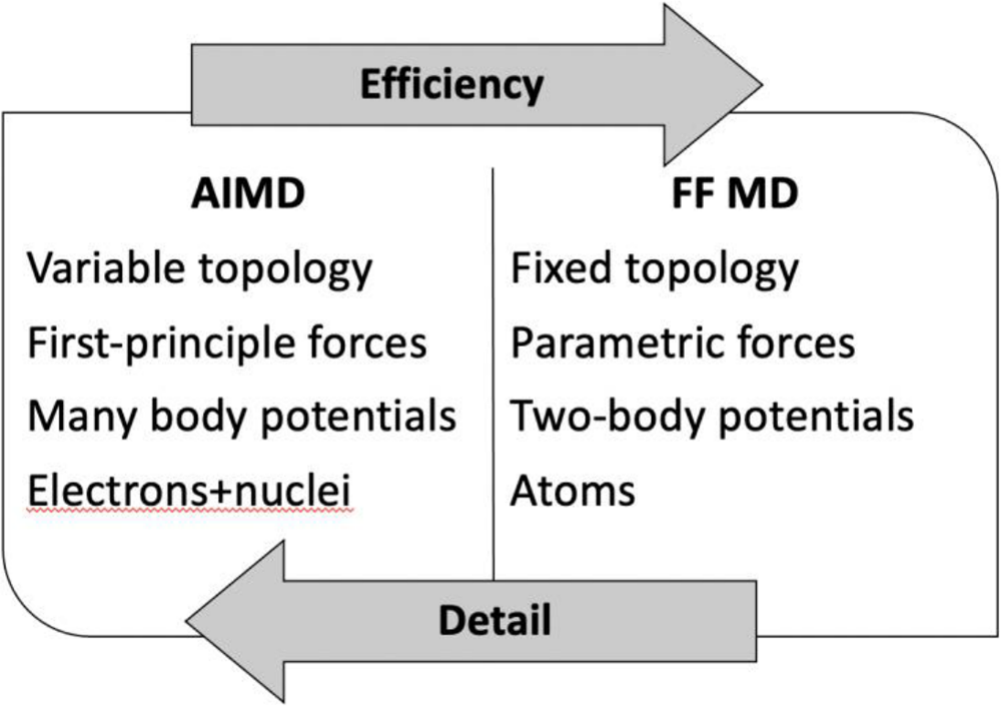
This sketch illustrates the main features of the two different ways of implementing MD in the typical setups of ILs theorical studies.

MD can be performed in various ways, the most common being ab initio MD, classical or force-field based MD and coarse-grained MD. The latter^53,54^ remains outside the scope of the present paper and we defer the reader to the specialized literature. The remaining two variants of MD have both advantages and drawbacks. We summarize them with the scheme of Figure 2.

AIMD is based on the simultaneous treatment of electrons and nuclei. The forces acting upon the nuclei are calculated via a solution of the electronic Schrödinger equation (typically with DFT) and differentiating the resulting electronic energy. The chemical topology (i.e., the presence of certain chemical bonds and the coordination of atoms) emerges naturally from the electronic structure and is not fixed. It is ideal to study the dynamic of chemical reactions, or any other bond breaking/forming process and it includes all nuclear many body potentials. It is extremely computationally expensive, and its applications are limited to a system with thousand atoms or less and for processes acting on a scale of picoseconds.

Classical MD, on the other hand, is much cheaper computationally, but the electrons disappear, the nuclei are blended into atoms, and the potential acting between them is parametric and (for the large part) made only by two-body terms. Such a simplified expression of the interatomic potential completely bypasses the need to evaluate forces using explicit electrons and results in a performance gain of several orders of magnitude with respect to ab initio methods. Typical systems for classical MD are well into the nanometric scale and in the microsecond regime.

Introducing (partially) many-body effects in classical MD is possible by using the so-called “polarizable” force fields (*vide infra*) that include electrostatic effects due to induced dipoles.^55−58^ In classical MD, the chemical topology of bonds is fixed and decided *a priori*. This last condition can be relaxed using special (“reactive”) force fields, that allow a change in atomic coordination, but have only seldom been applied to ILs.^59^

**Figure 3:**
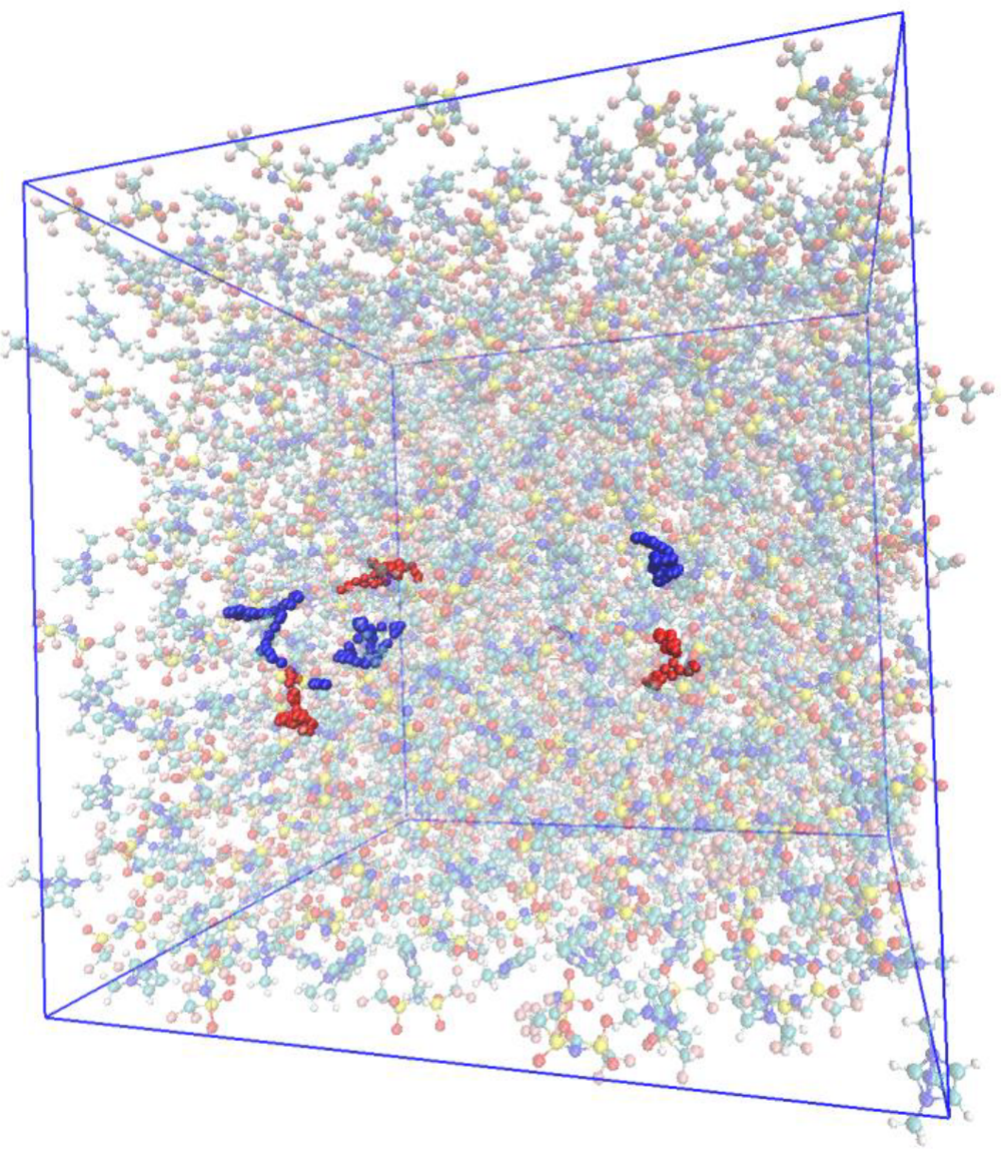
Exemplar results from a classical MD simulation of [MMIm][Tf2N] that illustrate the sluggish motion of molecular ions on a timespan of 1 ns. Against a shaded background, I have highlighted six ions (three cations in blue and three anions in red) and shown their centers of mass motion as a series of dots.

In order to realistically simulate a chemical event using MD one should, in principle, run the simulation long enough to see that event occurring on a statistically significant scale. In the case of ILs the sampling of the phase space for obtaining meaningful thermodynamics averages is a challenge per se. ILs are extremely viscous^60^ due to strong electrostatic forces between the molecular components, and collecting data with MD can prove frustrating. An idea of the sluggish motion of the centers of mass of molecular ions can be had by looking at Figure 3 that contains, for the sake of providing an example, data extracted from a simulation done with classical MD on a traditional IL. There I report 1 ns of motions of the centers of mass of 3 selected 1,3-dimethylimidazolium cations (blue) and 3 Tf_2_N anions (red) against the background (in shaded colors) of the other ions. In 1 ns, each ion does little more than oscillating around its initial position (the side of the cube is 60 Å wide), and it is clear that describing ionic motions needs very long time scales.

Such example should illustrate how properties that are related to the ionic motion (ionic diffusion, ligand exchange, rearrangements of solvation shells etc.) requires extreme simulation times that lie well beyond the present limits of methods based on a detailed calculation of the forces (ab initio MD, Figure 2, left). In other words, the more accurate the interactions, the less likely is to sample the time scales necessary to converge the desired property. To access long time scales, one has necessarily to resort to a simplified form of the interaction potentials, hence, to force fields-based methods. As I will detail below, the main challenge in performing simulations of ionic liquids stems from this apparent irremediable juxtaposition of accuracy vs time scale.

The problem lies in the fact that the growing use of ILs in complex technological setups that are beyond their simple use as bulk solvents (protein extraction, antimicrobic agents, energy storage devices, catalysis, advanced materials, etc.) already requires, and will do so even more in the future, an increasingly detailed description of their interaction and dynamic that goes well beyond classical MD models. The knowledge of these details has a cost that is linked to the long simulation times and that, at the moment, has to be paid in terms of computational resources. A great effort has been produced in the past and is presently put forward by the computational community in terms of optimizing and improving current algorithms and procedure to achieve the objective of allowing more and more detailed and reliable simulation techniques to become the de facto standard for the description of these systems.

## 4 Increasing Details in Classical MD: Polarization and Charge Transfer

It is well-known to the ILs community that, due to ionic nature of the materials, polarization is a crucial phenomenon that should be considered in the simulations. In nonpolarizable MD simulations, the atoms of a molecule interact with the atoms of another one via a potential made by three pieces: a repulsive, short-range part that defines the atomic volume; an attractive part that takes into account the van der Waals cohesive energy; and an electrostatic contribution due to the presence of a partial charge on each atom. In nonpolarizable MD, the partial atomic charges are fixed and depend on the nature of the atom and on the functional group to which it belongs. The atoms interact with each other solely via this potential; hence, traditional MD is entirely based on intermolecular forces that emerge from the sum of two body (atom–atom) interactions. When dealing with systems with partial charges significantly different from zero, the above approach begins to show some limitation. In the real system, induction effects becomes important, and the atomic charges fluctuate depending on the chemical surrounding of the atom. As an example, consider the solvation of a bivalent ion in water: the charge density of the ion induces dipoles on the water molecules that alter their charge distributions and that interact with each other. These interactions between the induced dipoles are many-body effects due to the simultaneous presence of at least three bodies (one inducer and two induced molecules). The potential used in traditional MD must be modified to account for the presence of these effects. This is the purpose of “polarizable” force fields. Using two different algorithmic procedures,^57,58^ they introduce a way to allow for charge fluctuations and to account for polarization and induction energy.

In practice, polarization effects are particularly important in fully ionized systems as ILs^55^ where these many-body interactions between the induced dipoles tend to reduce the electrostatic cohesive energy, thus explaining why polarizable force field lead to more reliable calculations of dynamical quantities such as fluidity, ionic mobility, and conductivities, all of which are heavily underestimated by fixed charge models.^55,61^

A part of the community is already involved in the setup of polarizable force fields which are presently recognized as an essential addition to traditional MD. A summary of these efforts has been recently reviewed^55^ and we only mention the recent availability of the CL&Pol^56,57,62^ and Amoeba-IL^58^ force fields. The two force fields are based on two entirely different algorithmic approaches, but both aims at becoming a sort of “standard” for polarizable simulations of ILs. It would be auspicious that in the future a wider dissemination of these force fields in the relevant community could provide the necessary validation to assess their potential and reliability.

As we mentioned above, the introduction of polarization is crucial whenever dynamics quantities are involved in the calculations. While structural and static properties can be easily obtained with a high degree of confidence also from nonpolarizable force fields, all those physical properties and phenomena that are related to the reorganization of the ILs molecules, hence friction, mobility, solvation, and transport, require the use of more sophisticated electrostatic models that go beyond the traditional fixed-charge schemes. Unfortunately, introducing polarization has a cost and the performance of MD degrades. An improvement of the algorithms is therefore mandatory to allow polarizable simulations to become the de facto standard method for producing reliable simulations in the ILs field.

Closely connected to polarization there is another peculiar phenomenon that affects the electrostatic of ILs and that is generally referred under the name of charge transfer. This is a quantum effects due to electronic delocalization across molecules. It consists of the fact that, when modeling ILs, the solution of the electronic SE (hence an ab initio approach) clearly indicates that the charge on each molecular ion is not unitary. In practice this means that the overall charge of cations and anions is lower than what is expected from the chemical structure. This effect is linked to the tight packing of the molecular ions caused by the strong electrostatic potential and to the transfer of significant portions of the electronic wave function across them.^63^

**Figure 4:**
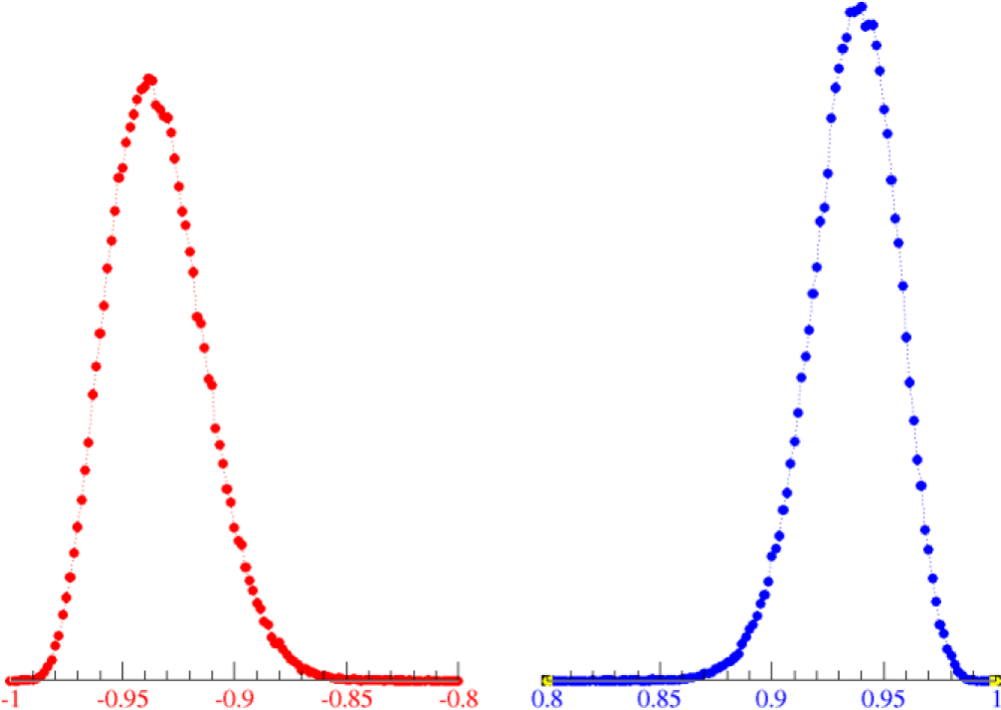
Charge distribution of the molecular ions in triethylammonium mesylate according to a semiempirical treatment of the electronic degree of freedom (DFTB) in a bulk simulation of 10 ps.

One example of this phenomenon is illustrated in Figure 4, where I report a calculation of the charge distributions of the two molecular ions of triethylammonium mesylate obtained through the approximate resolution of the electronic SE and sampled along 10 ps evolution. The net charge of the two ions (mesylate, negative, and triethylammonium, positive) is never ±1, but averages at ±0.93.

The charge transfer phenomenon is another important factor in explaining why the frictional properties of an ILs are overestimated by fixed charge models that assume an integer charge on the molecular ions. This effect is inherently quantum mechanical and cannot be easily taken into account using a model potential. Reducing the overall ionic charges by uniform scaling or introducing a dielectric screening are known and broadly used workarounds,^64^ but they are unsatisfactory from various points of view: the scaling factor is peculiar for each liquid and has to be known a priori from ab initio data or from reverse fitting experimental data; in addition, a uniform scaling does not produce a charge distribution such as that in Figure 4 but, rather, a single value of the ionic charges. More elaborate schemes to account for these effects are under study in force fields such as Amoeba+^65,66^ and in methods such as those based on the effective fragment potential.^67^

Presently, the best way to accurately take into account the phenomena of charge transfer is to perform AIMD, where the resolution of the SE at each step of the time-evolution ensures the calculation of forces that include polarization, many-body effects and, precisely, charge transfer. It is well-known, however, that AIMD require a huge computational effort that renders it, at the moment, impractical for the study of ILs over a time scale longer than few hundreds of picoseconds. We know that the bottleneck of the AIMD simulations is the resolution of the electronic SE and that any way of reducing the cost of this step opens the way for longer time scales and larger systems. One of the most promising way of implementing a reduction of the computational cost of AIMD is to resort to semiempirical approaches to solve the SE, hence drastically reducing the amount of linear algebra and iterations required at each step of the dynamics.

One of the approach that has been recently utilized in the ILs context is the density functional tight binding method (DFTB)^42,68−70^ that has shown good accuracy and performances. In its most recent implementation, the method is based on an expansion of the energy functional up to third order, it includes dispersion energy corrections and naturally produces realistic fluctuating atomic charges. It relies on the a priori knowledge of a set of parameters appearing in the energy expression that are calculated from atomic properties. This method is the one that produced the data reported in Figure 4.

It seems that semiempirical methods have been generally overlooked by the community as a possible tool to approach an almost first-principle description of ILs. While performance still is an issue for the wide applicability of these methods, there is a wide space for them in the near future especially in light of their ability to incorporate the electronic degrees of freedom in specific situations^71^ and to overcome several severe limit of classical MD (see Section 7) without being excessively expensive.

## 5 Modeling Collective Effects: Like-Charge Aggregation and Other Phenomena

It has been apparent from the beginning that, due to the variety of shapes and functional groups in the molecular ions, ILs can present a wide range of nanoscale structures whose nature is still not entirely understood. From the computational point of view, the emergence of these elusive, transient structures, which nevertheless leave their mark on the bulk liquid properties, is a fascinating, but difficult challenge. The nature of the conundrum is the following. Capturing the nature of these features at the molecular level using atomistic simulations involves operating with sizes large enough to accommodate these (often nanosized) structures for very long time scales.^72^ The problem is that to reach such regimes, computational efficiency requires the most simplified potential models (typically nonpolarizable force fields), but the lack of details in the potential might jeopardize the discovery of the driving force of the phenomena.

The presence of such nanostructures in the imidazolium-based generation of ILs, is clearly indicated by the appearance of low-*Q* peaks in the diffraction patterns.^34,73−75^ The aggregation and coalescence of alkyl groups in apolar domains is very likely at the origin of such spectral evidence. Computational studies, mostly based on classical MD in this context, have only provided indirect evidence about the nature of these domains, since the scales at which these aggregations occur are large both in terms of sizes and time. Only recently, more rigorous analysis have shed light on how these segregated domains of alkyl groups have a consequences on bulk properties such as structural relaxation and viscosities.^33,76^

In PILs the network of HBs represents an additional source of disorder and the formation of segregated apolar domains is often suppressed (for peculiar cases see refs (34 and 35)). However, even in the presence of conspicuous HBs, the formation of peculiar structures is not impossible. Our recent works on AAILs (rich in HBs)^77,78^ and the work of Ludwig and co-workers^36,37,39,40^ have shown that the presence of HBs can promote a different form of aggregation that defies our chemical intuition. This aggregation phenomena is due to like-charge interactions and seems to be peculiar of liquids rich in HBs. It consists of the formation of dimers or oligomers of ions of the same charge that are connected by strong HBs and where the expected Coulomb repulsion is weakened. The repulsive potential turns out to be mitigated by the charge delocalization in certain functional groups (for example carboxylates or sulfonates), by charge transfer which reduce the overall charge of the ions and by the electrostatic screening of the surrounding medium (polarization).

**Figure 5:**
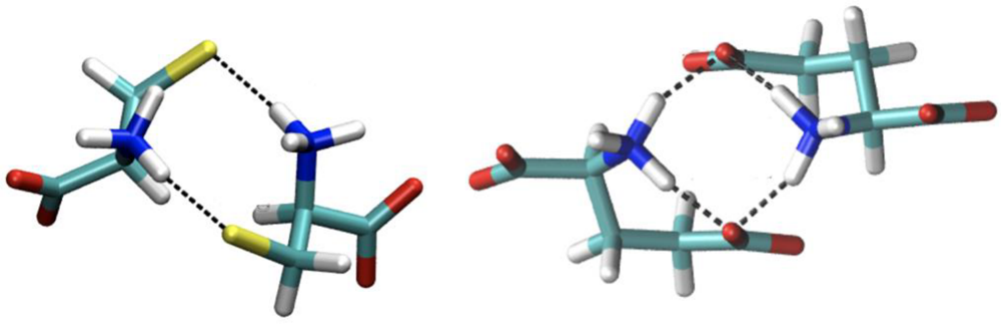
Lowest energy, optimized structures of the [Cys]_2_^2–^ (left) and [Glu]_2_^2–^ (right) dianionic dimers as obtained from ab initio calculations in the bulk phase. Reprinted with permission from ref (41). Copyright 2018 American Chemical Society.

For example, the anionic dimers [Cys]_2_^2–^ and [Glu]_2_^2–^, shown in Figure 5, are surprisingly stable when immersed in the bulk and their binding energies are comparable to those of the cation/anion counterpart.^79^ This is completely counterintuitive.

To correctly account for such elusive aggregation phenomena the computational model must include all the effects that are responsible for them. A purely fixed charge model would eventually lead to the dissociation of like-charge aggregates under their mutual repulsion. It is evident from the cited literature, that an accurate modeling of polarization and charge transfer are both prerequisites to describe the transient existence of these like-charge aggregated domains and the peculiar role of strong HBs in this context.

## 6 Accounting for Variable Chemical Topology: Ionicity and Reactions

PILs, as discussed in Section 2, arise formally from and acid–base equilibrium reaction. When the equilibrium is not entirely shifted toward the ionic pair, the ensuing liquid is a mixture of a neutral (polar) phase and an ionic one. In this case the system can undergo phase separation to various extent and to evaporation of the polar volatile component.^80,81^

With the help of recent works,^82−84,69^ a more complex picture than expected is emerging. Several mixtures of acids and bases that were expected to form fully ionic liquids are instead partially neutral even when the difference of p*K*_a_ between the two components is large. In addition, the extent of ionization does not seem to be trivially connected to this difference, but rather to the self-solvation abilities of the liquid toward its own ion. This issue is known as the “ionicity problem”. Predicting from molecular properties the extent of equilibrium 1 in the bulk fluid is nontrivial, and further studies and simulations are needed in the near future to fully address the ionicity of PILs.

The coexistence of a neutral and ionic phase makes the computational treatment of these liquids lying at the border of the ionic-to-polar transition extremely difficult. This is not an issue that can be simply solved by focusing on acid/base pairs that give rise to a full ionization. There are, in fact, many instances where PILs are chosen because of their ability to sustain proton conductivity.^85−89^ Proton conduction is achieved by using specific mixtures with partially dissociated acids or by precisely exploiting equilibrium 1. In all these cases the fate of proton transfer reactions between the molecular ions or within a mixture must be properly described by the computational model.

The problem is that traditional MD (even the polarizable variant) are based on a fixed chemical topology where the H atoms (protons) are initially bound to one molecular entity and remain bound to it during the entire simulations. Hence, traditional MD is unable to account for the proton transfer process, proton conductivity, tautomerization reaction, etc. To explore the dynamics of mobile protons the only option is to abandon fixed topology. This can be achieved in two ways: the first is to adopt first-principles MD either ab initio or semiempirical, and the second is to use a force field where the coordination number of each atom is not fixed but changes in response to the chemical environment.

**Figure 6:**
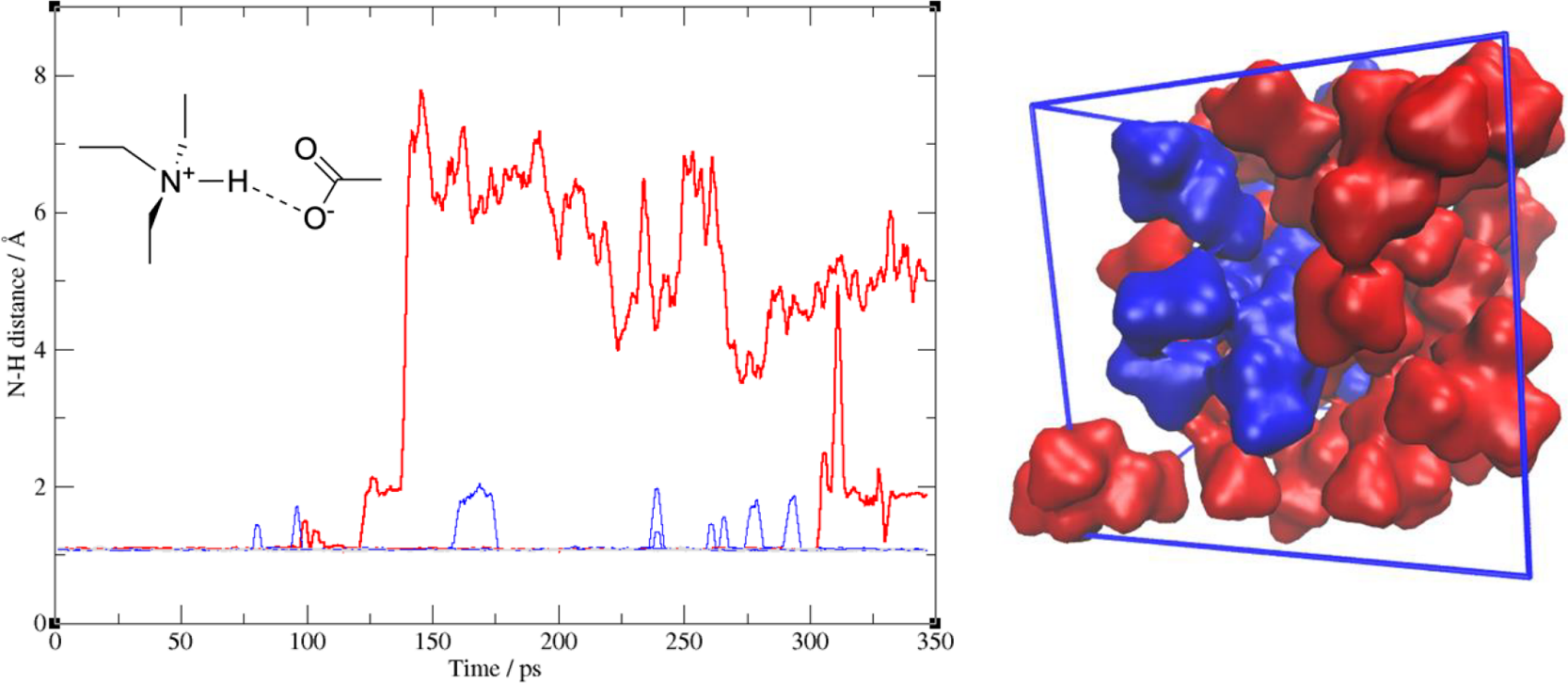
Left: Time evolution of the N–H distances of the triethylammonium ions in the bulk phase when coupled to acetate. The initial stage of proton migration from ammonium to acetate is illustrated by the two red trajectories. Right: The final state of the liquid—in red the neutral phase; in blue, the ionic one.

The former approach has been described above and, since chemical topology emerges naturally from the electronic SE, such method is, in principle, able to describe the evolution of any chemical reaction including proton transfer. To provide an example, we show in Figure 6 (left) the evolution of the N–H distances in the liquid triethylammonium acetate calculated using the DFTB method along 350 ps. The system at *t* = 0 is prepared as a fully ionic system (protons on the ammonium) and all N–H distances are around 1.1 Å. After 125 ps, a first proton hops onto one of the carboxylates neutralizing a pair of molecules (red trajectory). After 300 ps another one does the same thing. Along the dynamics one can also notice how several ammonium protons temporarily break the N–H bond (blue trajectories).

At the end of the proton migration process, the equilibrium state of the fluid should resemble the situation depicted in Figure 6 on the right. The red regions are the volumetric representation of the neutral phase, the blue one the aggregated region where the ionic species reside. It is clear from this simple illustration how chemical reactions and complex electrostatic forces do contribute to shape the unconventional nanoscopic structure of the fluid and of the aggregation/segregation phenomena within.

Most of the biocompatible, new generation ILs are obtained using amino acids anions and the cholinium cation. Choline is a very poor base and equilibrium 1 is entirely shifted to the right. This however does not mean that the problem of proton transfer is irrelevant for these liquids. For example, several amino acids have protic side chains (−SH, −PO_3_H_2_ or −COOH) that may lose the proton and lead to isomerization reactions that cause the appearance of zwitterionic-anionic forms of the amino acid. In addition, the spatial vicinity of anionic species due to anionic clustering (Section 6) makes the proton exchange between these protic groups and −NH_2_ very efficient. Hence, like in the case of incomplete proton transfer, the presence of additional (mobile) protons in AAILs could induce intra- and intermolecular isomerization reactions. For example, the singly deprotonated aspartate anion can exist in a set of almost isoenergetic isomers shown in Scheme 1. These three isomers are presumably in equilibrium through continuous and reversible proton transfer processes and their relative abundances can have a sizable effect on the dynamic and thermodynamic properties of the bulk phase.

Accounting for the presence of these phenomena in a simulation is not easy. The problem is that the typical time scales for establishing proton transfer equilibria can be very long, especially in highly viscous systems as PILs. While semiempirical and ab initio MD, are viable options to describe such equilibria, their typical time scales might lie well outside the reach of the current implementations of these methods. In other words, due the high viscosities of the fluids, simulations long enough to reach a steady state for the isomerization reactions are impractical and massively time-consuming. This means that, at present, the only methods that are appropriate to the study of proton conduction or partial ionization are still far from being able to provide a complete description of these processes simply because they cannot be easily extended to the time scales necessary to reach thermodynamic equilibrium.

When chemical reactions are involved, a more efficient approach is available and consists of using force fields where the atomic coordination numbers are variable and depend on the chemical surrounding. Such possibility is, however, still unexplored in the realm of ILs, and its use is certainly complicated by the fact that the transfer of protons also changes the charge and geometry of the molecular ion. While such force fields exists in other contexts,^90,91^ as far as I know, they have never been applied to proton transfer in ILs. The only case that I am aware of is the simulation of CO_2_ absorption by the AAIL [P_4444_][Gly].^59^ The problems of such an approach are that one must know in advance all possible reactive events to provide a realistic parametrization and that a large number of ab initio calculations are necessary for the characterization of these events.

## 7 Perspectives for the Near Future

Computational modeling of complex materials has become a key ingredient of research. The possibility of screening compounds and substance for a given desired property beforehand experimental investigation is an obvious benefit of the use of simulations. Beyond this, the growth in the availability of computer simulations coupled with improved algorithms and with the surge of hardware technical advances have allowed in the past few decades an unprecedented number of researchers to dedicate their work to the fundamental understanding of the phenomena that characterize new materials such as ILs. Despite these advances, though, many challenges still must be faced, some of which I have reviewed in this paper. The most prominent are those connected to the reliability of the models of the intermolecular potentials and to the applications of well-established techniques in situations where additional complications due to chemistry arise. In this context, I have laid out the status of computational research. What is to come is more difficult to foresee, but a glimpse of future development can be, nevertheless, attempted.

Accessing to reliable methods for the modeling of proton transfer dynamics in ILs is an important question: not only because ILs are currently widely employed as prototypical conductive medium in energy devices but also because their interaction with other molecules would necessarily depend on their acidity/basicity properties. I am especially thinking about the interaction of ILs (and their subgroups based on biocompatible molecular ions) with biomolecules and, ultimately, with living organisms. If the use of ILs should become so pervasive as the current research may induce one to foresee, it is evident that a proper modeling of their toxicity, biocompatibility, and biodegradability will be mandatory in the near future. While experimental evidence is accumulating,^23,92,93^ simulations and computational approaches about this subject are still scarce.^93−95^

In the context of proton mobility, a substantial advance in the current simulation techniques is necessary and 2-fold: on the one hand, the development of reliable potential models (semiempirical and reactive force fields) is stringent; on the other, the need of accounting for quantum nuclear effects might become an additional challenge.^7^ Tunnelling and other phenomena connected to the quantum nature of the light nuclei (protons) can be relevant to understand the biochemical activity of HB-dominated liquids such as AAILs.

For what concerns the outstanding problems of the computational cost, apart from improving algorithms and specific codes, the research could take advantage of relatively new procedures that exploit recent application of the chemoinformatic models to ILs. These statistical models allow one to trace the structure–activity relationship (SAR, QSAR, etc.)^96−100^ avoiding altogether the need to perform actual time-consuming MD and use relatively cheap ab initio calculations on isolated constituents.^101^

## References

[ref1] Park J. W.; Al-Saadon R.; MacLeod M. K.; Shiozaki T.; Vlaisavljevich B. Multireference Electron Correlation Methods: Journeys along Potential Energy Surfaces. Chem. Rev. 2020, 120, 5878–5909. 10.1021/acs.chemrev.9b00496. 32239929

[ref2] Bartlett R. J.; Musiał M. Coupled-Cluster Theory in Quantum Chemistry. Rev. Mod. Phys. 2007, 79, 291–352. 10.1103/RevModPhys.79.291.

[ref3] Becke A. D. Perspective: Fifty Years of Density-Functional Theory in Chemical Physics. J. Chem. Phys. 2014, 140, 18A301 10.1063/1.4869598. 24832308

[ref4] Regenmortel M. H. V. V. Reductionism and Complexity in Molecular Biology: Scientists Now Have the Tools to Unravel Biological Complexity and Overcome the Limitations of Reductionism. EMBO Rep. 2004, 5, 1016–1020. 10.1038/sj.embor.7400284. 15520799 PMC1299179

[ref5] Ingólfsson H. I.; Lopez C. A.; Uusitalo J. J.; de Jong D. H.; Gopal S. M.; Periole X.; Marrink S. J. The Power of Coarse Graining in Biomolecular Simulations: The Power of Coarse Graining in Biomolecular Simulations. Wiley Interdiscip. Rev. Comput. Mol. Sci. 2014, 4, 225–248. 10.1002/wcms.1169. 25309628 PMC4171755

[ref6] Brooks C. L.; Case D. A.; Plimpton S.; Roux B.; van der Spoel D.; Tajkhorshid E. Classical Molecular Dynamics. J. Chem. Phys. 2021, 154, 100401 10.1063/5.0045455. 33722022

[ref7] Markland T. E.; Ceriotti M. Nuclear Quantum Effects Enter the Mainstream. Nat. Rev. Chem. 2018, 2, 0109 10.1038/s41570-017-0109.

[ref8] Tuckerman M. E. *Ab Initio* Molecular Dynamics: Basic Concepts, Current Trends and Novel Applications. J. Phys.: Condens. Matter 2002, 14, R1297–R1355. 10.1088/0953-8984/14/50/202.

[ref9] Computational Molecular Dynamics: Challenges, Methods, Ideas: Proceedings of the 2nd International Symposium on Algorithms for Macromolecular Modelling, Berlin, May 21–24, 1997; Hermans J.; Leimkuhler B.; Reich S.; Mark A. E.; Deuflhard P.; Skeel R. D., Eds.; Springer: Berlin and Heidelberg, Germany, 1999.

[ref10] Gerber R. B.; Shemesh D.; Varner M. E.; Kalinowski J.; Hirshberg B. Ab Initio and Semi-Empirical Molecular Dynamics Simulations of Chemical Reactions in Isolated Molecules and in Clusters. Phys. Chem. Chem. Phys. 2014, 16, 9760–9775. 10.1039/C3CP55239J. 24569494

[ref11] Nishizawa H.; Nishimura Y.; Kobayashi M.; Irle S.; Nakai H. Three Pillars for Achieving Quantum Mechanical Molecular Dynamics Simulations of Huge Systems: Divide-and-Conquer, Density-Functional Tight-Binding, and Massively Parallel Computation. J. Comput. Chem. 2016, 37, 1983–1992. 10.1002/jcc.24419. 27317328

[ref12] Stettner T.; Balducci A. Protic Ionic Liquids in Energy Storage Devices: Past, Present and Future Perspective. Energy Storage Mater. 2021, 40, 402–414. 10.1016/j.ensm.2021.04.036.

[ref13] Jónsson E. Ionic Liquids as Electrolytes for Energy Storage Applications – A Modelling Perspective. Energy Storage Mater. 2020, 25, 827–835. 10.1016/j.ensm.2019.08.030.

[ref14] Kobzar Y. L.; Fatyeyeva K. Ionic Liquids as Green and Sustainable Steel Corrosion Inhibitors: Recent Developments. Chem. Eng. J. 2021, 425, 131480 10.1016/j.cej.2021.131480.

[ref15] Xu P.; Liang S.; Zong M.-H.; Lou W.-Y. Ionic Liquids for Regulating Biocatalytic Process: Achievements and Perspectives. Biotechnol. Adv. 2021, 51, 107702 10.1016/j.biotechadv.2021.107702. 33515671

[ref16] Krishnan A.; Gopinath K. P.; Vo D.-V. N.; Malolan R.; Nagarajan V. M.; Arun J. Ionic Liquids, Deep Eutectic Solvents and Liquid Polymers as Green Solvents in Carbon Capture Technologies: A Review. Environ. Chem. Lett. 2020, 18, 2031–2054. 10.1007/s10311-020-01057-y.

[ref17] Paucar N. E.; Kiggins P.; Blad B.; De Jesus K.; Afrin F.; Pashikanti S.; Sharma K. Ionic Liquids for the Removal of Sulfur and Nitrogen Compounds in Fuels: A Review. Environ. Chem. Lett. 2021, 19, 1205–1228. 10.1007/s10311-020-01135-1.

[ref18] Liu F.; Yu J.; Qazi A. B.; Zhang L.; Liu X. Metal-Based Ionic Liquids in Oxidative Desulfurization: A Critical Review. Environ. Sci. Technol. 2021, 55, 1419–1435. 10.1021/acs.est.0c05855. 33433212

[ref19] Shaikh A. R.; Ashraf M.; AlMayef T.; Chawla M.; Poater A.; Cavallo L. Amino Acid Ionic Liquids as Potential Candidates for CO2 Capture: Combined Density Functional Theory and Molecular Dynamics Simulations. Chem. Phys. Lett. 2020, 745, 137239 10.1016/j.cplett.2020.137239.

[ref20] Alizadeh V.; Esser L.; Kirchner B. How Is CO _2_ Absorbed into a Deep Eutectic Solvent?. J. Chem. Phys. 2021, 154, 094503 10.1063/5.0038093. 33685170

[ref21] Wang L.; Zhang Y.; Liu Y.; Xie H.; Xu Y.; Wei J. SO2 Absorption in Pure Ionic Liquids: Solubility and Functionalization. J. Hazard. Mater. 2020, 392, 122504 10.1016/j.jhazmat.2020.122504. 32208319

[ref22] Zubeltzu J.; Formoso E.; Rezabal E. Lignin Solvation by Ionic Liquids: The Role of Cation. J. Mol. Liq. 2020, 303, 112588 10.1016/j.molliq.2020.112588.

[ref23] Kumari P.; Pillai V. V. S.; Benedetto A. Mechanisms of Action of Ionic Liquids on Living Cells: The State of the Art. Biophys. Rev. 2020, 12, 1187–1215. 10.1007/s12551-020-00754-w. 32936423 PMC7575683

[ref24] Cho C.-W.; Pham T. P. T.; Zhao Y.; Stolte S.; Yun Y.-S. Review of the Toxic Effects of Ionic Liquids. Sci. Total Environ. 2021, 786, 147309 10.1016/j.scitotenv.2021.147309. 33975102

[ref25] Chen Y.; Mu T. Revisiting Greenness of Ionic Liquids and Deep Eutectic Solvents. Green Chem. Eng. 2021, 2, 174–186. 10.1016/j.gce.2021.01.004.

[ref26] Magina S.; Barros-Timmons A.; Ventura S. P. M.; Evtuguin D. V. Evaluating the Hazardous Impact of Ionic Liquids – Challenges and Opportunities. J. Hazard. Mater. 2021, 412, 125215 10.1016/j.jhazmat.2021.125215. 33951860

[ref27] Le Donne A.; Bodo E. Cholinium Amino Acid-Based Ionic Liquids. Biophys. Rev. 2021, 13, 147 10.1007/s12551-021-00782-0. 33747249 PMC7930144

[ref28] Gontrani L. Choline-Amino Acid Ionic Liquids: Past and Recent Achievements about the Structure and Properties of These Really “Green” Chemicals. Biophys. Rev. 2018, 10, 873–880. 10.1007/s12551-018-0420-9. 29687272 PMC5988634

[ref29] Gomes J. M.; Silva S. S.; Reis R. L. Biocompatible Ionic Liquids: Fundamental Behaviours and Applications. Chem. Soc. Rev. 2019, 48, 4317–4335. 10.1039/C9CS00016J. 31225558

[ref30] Wang Y.-L.; Li B.; Sarman S.; Mocci F.; Lu Z.-Y.; Yuan J.; Laaksonen A.; Fayer M. D. Microstructural and Dynamical Heterogeneities in Ionic Liquids. Chem. Rev. 2020, 120, 5798–5877. 10.1021/acs.chemrev.9b00693. 32292036 PMC7349628

[ref31] Dhattarwal H. S.; Kashyap H. K. Unique and Generic Structural Features of Cholinium Amino Acid-Based Biocompatible Ionic Liquids. Phys. Chem. Chem. Phys. 2021, 23, 10662–10669. 10.1039/D1CP00937K. 33908525

[ref32] Busato M.; Di Lisio V.; Del Giudice A.; Tomai P.; Migliorati V.; Galantini L.; Gentili A.; Martinelli A.; D’Angelo P. Transition from Molecular- to Nano-Scale Segregation in a Deep Eutectic Solvent - Water Mixture. J. Mol. Liq. 2021, 331, 115747 10.1016/j.molliq.2021.115747.

[ref33] Amith W. D.; Araque J. C.; Margulis C. J. A Pictorial View of Viscosity in Ionic Liquids and the Link to Nanostructural Heterogeneity. J. Phys. Chem. Lett. 2020, 11, 2062–2066. 10.1021/acs.jpclett.0c00170. 32079397

[ref34] Miao S.; Wood J.; Jiang H. J.; Imberti S.; Atkin R.; Warr G. Unusual Origin of Choline Phenylalaninate Ionic Liquid Nanostructure. J. Mol. Liq. 2020, 319, 114327 10.1016/j.molliq.2020.114327.

[ref35] Miao S.; Atkin R.; Warr G. G. Amphiphilic Nanostructure in Choline Carboxylate and Amino Acid Ionic Liquids and Solutions. Phys. Chem. Chem. Phys. 2020, 22, 3490–3498. 10.1039/C9CP06752C. 31990285

[ref36] Niemann T.; Neumann J.; Stange P.; Gärtner S.; Youngs T. G. A.; Paschek D.; Warr G. G.; Atkin R.; Ludwig R. The Double-Faced Nature of Hydrogen Bonding in Hydroxy-Functionalized Ionic Liquids Shown by Neutron Diffraction and Molecular Dynamics Simulations. Angew. Chem., Int. Ed. 2019, 58, 12887–12892. 10.1002/anie.201904712. 31177605

[ref37] Khudozhitkov A. E.; Neumann J.; Niemann T.; Zaitsau D.; Stange P.; Paschek D.; Stepanov A. G.; Kolokolov D. I.; Ludwig R. Hydrogen Bonding Between Ions of Like Charge in Ionic Liquids Characterized by NMR Deuteron Quadrupole Coupling Constants—Comparison with Salt Bridges and Molecular Systems. Angew. Chem., Int. Ed. 2019, 58, 17863–17871. 10.1002/anie.201912476. PMC6899581 31588622

[ref38] Neumann J.; Ludwig R.; Paschek D. Hydrogen Bonds between Ions of Opposite and Like Charge in Hydroxyl-Functionalized Ionic Liquids: An Exhaustive Examination of the Interplay between Global and Local Motions and Intermolecular Hydrogen Bond Lifetimes and Kinetics. J. Phys. Chem. B 2021, 125, 5132–5144. 10.1021/acs.jpcb.1c02756. 33971719

[ref39] Niemann T.; Zaitsau D. H.; Strate A.; Stange P.; Ludwig R. Controlling “like–Likes–like” Charge Attraction in Hydroxy-Functionalized Ionic Liquids by Polarizability of the Cations, Interaction Strength of the Anions and Varying Alkyl Chain Length. Phys. Chem. Chem. Phys. 2020, 22, 2763–2774. 10.1039/C9CP06481H. 31951236

[ref40] Niemann T.; Zaitsau D.; Strate A.; Villinger A.; Ludwig R. Cationic Clustering Influences the Phase Behaviour of Ionic Liquids. Sci. Rep. 2018, 8, 14753 10.1038/s41598-018-33176-6. 30283059 PMC6170405

[ref41] Le Donne A.; Adenusi H.; Porcelli F.; Bodo E. Hydrogen Bonding as a Clustering Agent in Protic Ionic Liquids: Like-Charge vs Opposite-Charge Dimer Formation. ACS Omega 2018, 3, 10589–10600. 10.1021/acsomega.8b01615. 31459182 PMC6645488

[ref42] Adenusi H.; Le Donne A.; Porcelli F.; Bodo E. Ab Initio Molecular Dynamics Study of Phospho-Amino Acid-Based Ionic Liquids: Formation of Zwitterionic Anions in the Presence of Acidic Side Chains. J. Phys. Chem. B 2020, 124, 1955–1964. 10.1021/acs.jpcb.9b09703. 32037824 PMC7997564

[ref43] Zandu S. K.; Chopra H.; Singh I. Ionic Liquids for Therapeutic and Drug Delivery Applications. Curr. Drug Res. Rev. 2020, 12, 26–41. 10.2174/2589977511666191125103338. 31763972

[ref44] Md Moshikur R.; Chowdhury R.; Moniruzzaman M.; Goto M. Biocompatible Ionic Liquids and Their Applications in Pharmaceutics. Green Chem. 2020, 22, 8116–8139. 10.1039/D0GC02387F.

[ref45] Saptal V. B.; Bhanage B. M. Bifunctional Ionic Liquids Derived from Biorenewable Sources as Sustainable Catalysts for Fixation of Carbon Dioxide. ChemSusChem 2017, 10, 1145–1151. 10.1002/cssc.201601228. 27763737

[ref46] Baaqel H.; Díaz I.; Tulus V.; Chachuat B.; Guillén-Gosálbez G.; Hallett J. P. Role of Life-Cycle Externalities in the Valuation of Protic Ionic Liquids – a Case Study in Biomass Pretreatment Solvents. Green Chem. 2020, 22, 3132–3140. 10.1039/D0GC00058B.

[ref47] Asim A. M.; Uroos M.; Muhammad N. Extraction of Lignin and Quantitative Sugar Release from Biomass Using Efficient and Cost-Effective Pyridinium Protic Ionic Liquids. RSC Adv. 2020, 10, 44003–44014. 10.1039/D0RA09098K. PMC9058325 35517143

[ref48] Stettner T.; Lingua G.; Falco M.; Balducci A.; Gerbaldi C. Protic Ionic Liquids-Based Crosslinked Polymer Electrolytes: A New Class of Solid Electrolytes for Energy Storage Devices. Energy Technol. 2020, 8, 2000742 10.1002/ente.202000742.

[ref49] Gerlach P.; Burges R.; Lex-Balducci A.; Schubert U. S.; Balducci A. Aprotic and Protic Ionic Liquids as Electrolytes for Organic Radical Polymers. J. Electrochem. Soc. 2020, 167, 120546 10.1149/1945-7111/abb382.

[ref50] Manjunatha L.; Takamatsu H.; Cannon J. J. Atomic-Level Breakdown of Green–Kubo Relations Provides New Insight into the Mechanisms of Thermal Conduction. Sci. Rep. 2021, 11, 5597 10.1038/s41598-021-84446-9. 33692393 PMC7970939

[ref51] Reddy Th. D. N.; Mallik B. S. Connecting Correlated and Uncorrelated Transport to Dynamics of Ionic Interactions in Cyclic Ammonium-Based Ionic Liquids. J. Phys. Chem. B 2020, 124, 6813–6824. 10.1021/acs.jpcb.0c00577. 32644816

[ref52] Atamas N.; Yablochkova K. S.; Lazarenko M. M. Microscopic Dynamics and the Dynamic Heterogeneity of Motion of Polar Molecules in Ionic Liquids. J. Mol. Liq. 2021, 332, 115900 10.1016/j.molliq.2021.115900.

[ref53] Bastos H.; Bento R.; Schaeffer N.; Coutinho J. A. P.; Pérez-Sánchez G. Using Coarse-Grained Molecular Dynamics to Rationalize Biomolecule Solubilization Mechanisms in Ionic Liquid-Based Colloidal Systems. Phys. Chem. Chem. Phys. 2020, 22, 24771–24783. 10.1039/D0CP04942E. 33107535

[ref54] Wang Y.-L.; Li B.; Laaksonen A. Coarse-Grained Simulations of Ionic Liquid Materials: From Monomeric Ionic Liquids to Ionic Liquid Crystals and Polymeric Ionic Liquids. Phys. Chem. Chem. Phys. 2021, 23, 19435–19456. 10.1039/D1CP02662C. 34524303

[ref55] Bedrov D.; Piquemal J.-P.; Borodin O.; MacKerell A. D.; Roux B.; Schröder C. Molecular Dynamics Simulations of Ionic Liquids and Electrolytes Using Polarizable Force Fields. Chem. Rev. 2019, 119, 7940–7995. 10.1021/acs.chemrev.8b00763. 31141351 PMC6620131

[ref56] Goloviznina K.; Gong Z.; Padua A. A. H. The CL&Pol Polarizable Force Field for the Simulation of Ionic Liquids and Eutectic Solvents. WIREs Comput. Mol. Sci. 2021, e1572, 1–16. 10.1002/wcms.1572. 33555860

[ref57] Goloviznina K.; Canongia Lopes J. N.; Costa Gomes M.; Pádua A. A. H. Transferable, Polarizable Force Field for Ionic Liquids. J. Chem. Theory Comput. 2019, 15, 5858–5871. 10.1021/acs.jctc.9b00689. 31525922

[ref58] Vázquez-Montelongo E. A.; Vázquez-Cervantes J. E.; Cisneros G. A. Current Status of AMOEBA–IL: A Multipolar/Polarizable Force Field for Ionic Liquids. Int. J. Mol. Sci. 2020, 21, 697 10.3390/ijms21030697. PMC7037047 31973103

[ref59] Zhang B.; van Duin A. C. T.; Johnson J. K. Development of a ReaxFF Reactive Force Field for Tetrabutylphosphonium Glycinate/CO _2_ Mixtures. J. Phys. Chem. B 2014, 118, 12008–12016. 10.1021/jp5054277. 25285669

[ref60] Bouarab A. F.; Harvey J.-P.; Robelin C. Viscosity Models for Ionic Liquids and Their Mixtures. Phys. Chem. Chem. Phys. 2021, 23, 733–752. 10.1039/D0CP05787H. 33427279

[ref61] Tu Y.-J.; Lin Z.; Allen M. J.; Cisneros G. A. Molecular Dynamics Investigation of Water-Exchange Reactions on Lanthanide Ions in Water/1-Ethyl-3-Methylimidazolium Trifluoromethylsufate ([EMIm][OTf]). J. Chem. Phys. 2018, 148, 024503 10.1063/1.4997008. 29331119

[ref62] Goloviznina K.; Gong Z.; Costa Gomes M. F.; Pádua A. A. H. Extension of the CL&Pol Polarizable Force Field to Electrolytes, Protic Ionic Liquids, and Deep Eutectic Solvents. J. Chem. Theory Comput. 2021, 17, 1606–1617. 10.1021/acs.jctc.0c01002. 33555860

[ref63] Hollóczki O.; Malberg F.; Welton T.; Kirchner B. On the Origin of Ionicity in Ionic Liquids. Ion Pairing versus Charge Transfer. Phys. Chem. Chem. Phys. 2014, 16, 16880–16890. 10.1039/C4CP01177E. 25012230

[ref64] Schröder C. Comparing Reduced Partial Charge Models with Polarizable Simulations of Ionic Liquids. Phys. Chem. Chem. Phys. 2012, 14, 3089 10.1039/c2cp23329k. 22287020 PMC7613810

[ref65] Liu C.; Piquemal J.-P.; Ren P. AMOEBA+ Classical Potential for Modeling Molecular Interactions. J. Chem. Theory Comput. 2019, 15, 4122–4139. 10.1021/acs.jctc.9b00261. 31136175 PMC6615954

[ref66] Liu C.; Piquemal J.-P.; Ren P. Implementation of Geometry-Dependent Charge Flux into the Polarizable AMOEBA+ Potential. J. Phys. Chem. Lett. 2020, 11, 419–426. 10.1021/acs.jpclett.9b03489. 31865706 PMC7384396

[ref67] Viquez Rojas C. I.; Slipchenko L. V. Exchange Repulsion in Quantum Mechanical/Effective Fragment Potential Excitation Energies: Beyond Polarizable Embedding. J. Chem. Theory Comput. 2020, 16, 6408–6417. 10.1021/acs.jctc.9b01156. 32786899

[ref68] Gaus M.; Cui Q.; Elstner M. DFTB3: Extension of the Self-Consistent-Charge Density-Functional Tight-Binding Method (SCC-DFTB). J. Chem. Theory Comput. 2011, 7, 931–948. 10.1021/ct100684s. PMC3509502 23204947

[ref69] Bodo E.; Bonomo M.; Mariani A. Assessing the Structure of Protic Ionic Liquids Based on Triethylammonium and Organic Acid Anions. J. Phys. Chem. B 2021, 125, 2781–2792. 10.1021/acs.jpcb.1c00249. 33719447 PMC8041315

[ref70] Cuny J.; Cerda Calatayud J.; Ansari N.; Hassanali A. A.; Rapacioli M.; Simon A. Simulation of Liquids with the Tight-Binding Density-Functional Approach and Improved Atomic Charges. J. Phys. Chem. B 2020, 124, 7421–7432. 10.1021/acs.jpcb.0c04167. 32696649

[ref71] Brehm M.; Sebastiani D. Simulating Structure and Dynamics in Small Droplets of 1-Ethyl-3-Methylimidazolium Acetate. J. Chem. Phys. 2018, 148, 193802 10.1063/1.5010342. 30307180

[ref72] Busato M.; Migliorati V.; Del Giudice A.; Di Lisio V.; Tomai P.; Gentili A.; D’Angelo P. Anatomy of a Deep Eutectic Solvent: Structural Properties of Choline Chloride : Sesamol 1 : 3 Compared to Reline. Phys. Chem. Chem. Phys. 2021, 23, 11746–11754. 10.1039/D1CP01105G. 33982713

[ref73] Reddy Th. D. N.; Mallik B. S. Heterogeneity in the Microstructure and Dynamics of Tetraalkylammonium Hydroxide Ionic Liquids: Insight from Classical Molecular Dynamics Simulations and Voronoi Tessellation Analysis. Phys. Chem. Chem. Phys. 2020, 22, 3466–3480. 10.1039/C9CP06796E. 31984978

[ref74] Jiang H. J.; Miao S.; Imberti S.; Simmons B. A.; Atkin R.; Warr G. G. Liquid Nanostructure of Choline Lysinate with Water and a Model Lignin Residue. Green Chem. 2021, 23, 856–866. 10.1039/D0GC03664A.

[ref75] Mariani A.; Innocenti A.; Varzi A.; Passerini S. On the Nanoscopic Structural Heterogeneity of Liquid *n* -Alkyl Carboxylic Acids. Phys. Chem. Chem. Phys. 2021, 23, 20282–20287. 10.1039/D1CP02846D. 34486605

[ref76] Amith W. D.; Araque J. C.; Margulis C. J. Relationship between the Relaxation of Ionic Liquid Structural Motifs and That of the Shear Viscosity. J. Phys. Chem. B 2021, 125, 6264–6271. 10.1021/acs.jpcb.1c03105. 34097825 PMC8279556

[ref77] Le Donne A.; Adenusi H.; Porcelli F.; Bodo E. Structural Features of Cholinium Based Protic Ionic Liquids through Molecular Dynamics. J. Phys. Chem. B 2019, 123, 5568–5576. 10.1021/acs.jpcb.9b03314. 31185161

[ref78] Campetella M.; Le Donne A.; Daniele M.; Gontrani L.; Lupi S.; Bodo E.; Leonelli F. Hydrogen Bonding Features in Cholinium-Based Protic Ionic Liquids from Molecular Dynamics Simulations. J. Phys. Chem. B 2018, 122, 2635–2645. 10.1021/acs.jpcb.7b12455. 29432015

[ref79] Le Donne A.; Adenusi H.; Porcelli F.; Bodo E. Hydrogen Bonding as a Clustering Agent in Protic Ionic Liquids: Like-Charge vs Opposite-Charge Dimer Formation. ACS Omega 2018, 3, 10589–10600. 10.1021/acsomega.8b01615. 31459182 PMC6645488

[ref80] Berton P.; Kelley S. P.; Wang H.; Rogers R. D. Elucidating the Triethylammonium Acetate System: Is It Molecular or Is It Ionic?. J. Mol. Liq. 2018, 269, 126–131. 10.1016/j.molliq.2018.08.006.

[ref81] Bodo E. Structural Features of Triethylammonium Acetate through Molecular Dynamics. Molecules 2020, 25, 1432 10.3390/molecules25061432. PMC7146455 32245229

[ref82] Nasrabadi A. T.; Gelb L. D. How Proton Transfer Equilibria Influence Ionic Liquid Properties: Molecular Simulations of Alkylammonium Acetates. J. Phys. Chem. B 2018, 122, 5961–5971. 10.1021/acs.jpcb.8b01631. 29750530

[ref83] Miran M. S.; Hoque M.; Yasuda T.; Tsuzuki S.; Ueno K.; Watanabe M. Key Factor Governing the Physicochemical Properties and Extent of Proton Transfer in Protic Ionic Liquids: ΔpKa or Chemical Structure?. Phys. Chem. Chem. Phys. 2019, 21, 418–426. 10.1039/C8CP06973E. 30534757

[ref84] Chen K.; Wang Y.; Yao J.; Li H. Equilibrium in Protic Ionic Liquids: The Degree of Proton Transfer and Thermodynamic Properties. J. Phys. Chem. B 2018, 122, 309–315. 10.1021/acs.jpcb.7b10671. 29232135

[ref85] Watanabe H.; Umecky T.; Arai N.; Nazet A.; Takamuku T.; Harris K. R.; Kameda Y.; Buchner R.; Umebayashi Y. Possible Proton Conduction Mechanism in Pseudo-Protic Ionic Liquids: A Concept of Specific Proton Conduction. J. Phys. Chem. B 2019, 123, 6244–6252. 10.1021/acs.jpcb.9b03185. 31251059

[ref86] Watanabe H.; Arai N.; Kameda Y.; Buchner R.; Umebayashi Y. Effect of Brønsted Acidity on Ion Conduction in Fluorinated Acetic Acid and *N* -Methylimidazole Equimolar Mixtures as Pseudo-Protic Ionic Liquids. J. Phys. Chem. B 2020, 124, 11157–11164. 10.1021/acs.jpcb.0c07706. 33198463

[ref87] Vázquez-Fernández I.; Raghibi M.; Bouzina A.; Timperman L.; Bigarré J.; Anouti M. Protic Ionic Liquids/Poly(Vinylidene Fluoride) Composite Membranes for Fuel Cell Application. J. Energy Chem. 2021, 53, 197–207. 10.1016/j.jechem.2020.04.022.

[ref88] Rao J.; Wang X.; Yunis R.; Ranganathan V.; Howlett P. C.; MacFarlane D. R.; Forsyth M.; Zhu H. A Novel Proton Conducting Ionogel Electrolyte Based on Poly(Ionic Liquids) and Protic Ionic Liquid. Electrochim. Acta 2020, 346, 136224 10.1016/j.electacta.2020.136224.

[ref89] Shmukler L. E.; Gruzdev M. S.; Kudryakova N. O.; Fadeeva Y. A.; Kolker A. M.; Safonova L. P. Triethylammonium-Based Protic Ionic Liquids with Sulfonic Acids: Phase Behavior and Electrochemistry. J. Mol. Liq. 2018, 266, 139–146. 10.1016/j.molliq.2018.06.059.

[ref90] Senftle T. P.; Hong S.; Islam M. M.; Kylasa S. B.; Zheng Y.; Shin Y. K.; Junkermeier C.; Engel-Herbert R.; Janik M. J.; Aktulga H. M.; Verstraelen T.; Grama A.; van Duin A. C. T. The ReaxFF Reactive Force-Field: Development, Applications and Future Directions. Npj Comput. Mater. 2016, 2, 15011 10.1038/npjcompumats.2015.11.

[ref91] van Duin A. C. T.; Zou C.; Joshi K.; Bryantsev V.; Goddard W. A. CHAPTER 6. A Reaxff Reactive Force-Field for Proton Transfer Reactions in Bulk Water and Its Applications to Heterogeneous Catalysis. In Catalysis Series; Asthagiri A., Janik M. J., Eds.; Royal Society of Chemistry: Cambridge, U.K., 2013; pp 223–243.

[ref92] Sivapragasam M.; Moniruzzaman M.; Goto M. An Overview on the Toxicological Properties of Ionic Liquids toward Microorganisms. Biotechnol. J. 2020, 15, 1900073 10.1002/biot.201900073. 31864234

[ref93] Bui-Le L.; Clarke C. J.; Bröhl A.; Brogan A. P. S.; Arpino J. A. J.; Polizzi K. M.; Hallett J. P. Revealing the Complexity of Ionic Liquid–Protein Interactions through a Multi-Technique Investigation. Commun. Chem. 2020, 3, 55 10.1038/s42004-020-0302-5. PMC9814843 36703418

[ref94] Wakayama R.; Uchiyama S.; Hall D. Ionic Liquids and Protein Folding—Old Tricks for New Solvents. Biophys. Rev. 2019, 11, 209–225. 10.1007/s12551-019-00509-2. 30888574 PMC6441443

[ref95] Lim G. S.; Klähn M. On the Stability of Proteins Solvated in Imidazolium-Based Ionic Liquids Studied with Replica Exchange Molecular Dynamics. J. Phys. Chem. B 2018, 122, 9274–9288. 10.1021/acs.jpcb.8b06452. 30192538

[ref96] Bernardes C. E. S.; Klimenko K.; Canongia Lopes J. N. Water Solubility Trends in Ionic Liquids: The Quantitative Structure–Property Relationship Model versus Molecular Dynamics. J. Phys. Chem. B 2021, 125, 11491–11497. 10.1021/acs.jpcb.1c06133. 34636241

[ref97] Lotfi S.; Ahmadi S.; Kumar P. A Hybrid Descriptor Based QSPR Model to Predict the Thermal Decomposition Temperature of Imidazolium Ionic Liquids Using Monte Carlo Approach. J. Mol. Liq. 2021, 338, 116465 10.1016/j.molliq.2021.116465.

[ref98] Yan J.; Yan X.; Hu S.; Zhu H.; Yan B. Comprehensive Interrogation on Acetylcholinesterase Inhibition by Ionic Liquids Using Machine Learning and Molecular Modeling. Environ. Sci. Technol. 2021, 55, 14720 10.1021/acs.est.1c02960. 34636548

[ref99] Ding Y.; Chen M.; Guo C.; Zhang P.; Wang J. Molecular Fingerprint-Based Machine Learning Assisted QSAR Model Development for Prediction of Ionic Liquid Properties. J. Mol. Liq. 2021, 326, 115212 10.1016/j.molliq.2020.115212.

[ref100] Abramenko N.; Kustov L.; Metelytsia L.; Kovalishyn V.; Tetko I.; Peijnenburg W. A Review of Recent Advances towards the Development of QSAR Models for Toxicity Assessment of Ionic Liquids. J. Hazard. Mater. 2020, 384, 121429 10.1016/j.jhazmat.2019.121429. 31732345

[ref101] Peng D.; Picchioni F. Prediction of Toxicity of Ionic Liquids Based on GC-COSMO Method. J. Hazard. Mater. 2020, 398, 122964 10.1016/j.jhazmat.2020.122964. 32768829

